# The Invertebrate Immunocyte: A Complex and Versatile Model for Immunological, Developmental, and Environmental Research

**DOI:** 10.3390/cells13242106

**Published:** 2024-12-19

**Authors:** Sandro Sacchi, Davide Malagoli, Nicola Franchi

**Affiliations:** 1 Department of Life Sciences, University of Modena and Reggio Emilia, 41125 Modena, Italy; sandro.sacchi@unimore.it (S.S.); nicola.franchi@unimore.it (N.F.); 2 National Biodiversity Future Center (NBFC), 90133 Palermo, Italy

**Keywords:** animal models, comparative immunology, eco-immunology, mollusk, *Pomacea canaliculata*

## Abstract

The knowledge of comparative and developmental immunobiology has grown over the years and has been strengthened by the contributions of multi-omics research. High-performance microscopy, flow cytometry, scRNA sequencing, and the increased capacity to handle complex data introduced by machine learning have allowed the uncovering of aspects of great complexity and diversity in invertebrate immunocytes, i.e., immune-related circulating cells, which until a few years ago could only be described in terms of morphology and basic cellular functions, such as phagocytosis or enzymatic activity. Today, invertebrate immunocytes are recognized as sophisticated biological entities, involved in host defense, stress response, wound healing, organ regeneration, but also in numerous functional aspects of organismal life not directly related to host defense, such as embryonic development, metamorphosis, and tissue homeostasis. The multiple functions of immunocytes do not always fit the description of invertebrate organisms as simplified biological systems compared to those represented by vertebrates. However, precisely the increasing complexity revealed by immunocytes makes invertebrate organisms increasingly suitable models for addressing biologically significant and specific questions, while continuing to present the undeniable advantages associated with their ethical and economic sustainability.

## 1. Introduction

Thanks to significant advances in immunological knowledge and experimental datasets, boosted by NGS technologies and deep learning-driven multi-omics analyses [1], the human immune system is now appreciated in its great complexity, and the challenges of developing adequate mathematical and experimental models to describe the intricate functions of the immune system, especially in organisms with adaptive immunity, are still ongoing [2]. This immune complexity, initially ascribed only to vertebrates, or more commonly, mammalian organisms, is also present in invertebrates, presenting new and unforeseen challenges in terms of the translation of knowledge gained from simpler models. The present review, while discouraging a perspective that describes the invertebrate immune system as a simplified model of vertebrate immunity, aims to summarize the evidence showing that even in anatomically simple organisms, the immune system is reliant on cells, i.e., immunocytes, that exhibit a previously unexpected functional complexity. It also aims to highlight the considerable benefits that can be derived from understanding this complexity.

## 2. Invertebrate Circulating Immune Cells: From Phagocytes to Immunocytes

From the early experiments on phagocytosis by Metchnikoff to the present day, a substantial portion of studies on the immune system of invertebrates has centered on the circulating phagocytic cell. Depending on its location and origin, this cell is commonly referred to as hemocyte [3] or coelomocyte [4,5,6]. These circulating cells have been classified and named in various ways, with terminologies and classification differing among the major taxa, including insects, crustaceans, mollusks, echinoderms, and tunicates, to name just a few examples [3]. Because invertebrates lack adaptive immunity based on lymphocyte-based processes such as affinity maturation and the presence of memory cells, invertebrate circulating cells have long been considered simpler versions of vertebrate phagocytes and, after the first hints of their complexity were observed, to the mammalian macrophage [7]. The expected roles of invertebrate hemocytes were primarily limited to defense against invading unicellular pathogens or multicellular parasites, before a broader immuno-neuroendocrine role was proposed [8]. This perspective has driven research in the direction of seeking conserved phenomena between invertebrate and vertebrate organisms to enhance our understanding of the innate component of the human immune system. The seminal studies that emerged from this perspective have significantly enhanced our understanding of the functioning of the human immune system and made a fundamental contribution to the discovery of the cooperation between innate and adaptive components of vertebrate immunity. This culminated in the co-award of the Nobel Prize in Physiology or Medicine for studies on innate immunity in *Drosophila melanogaster* [9].

**Figure 1:**
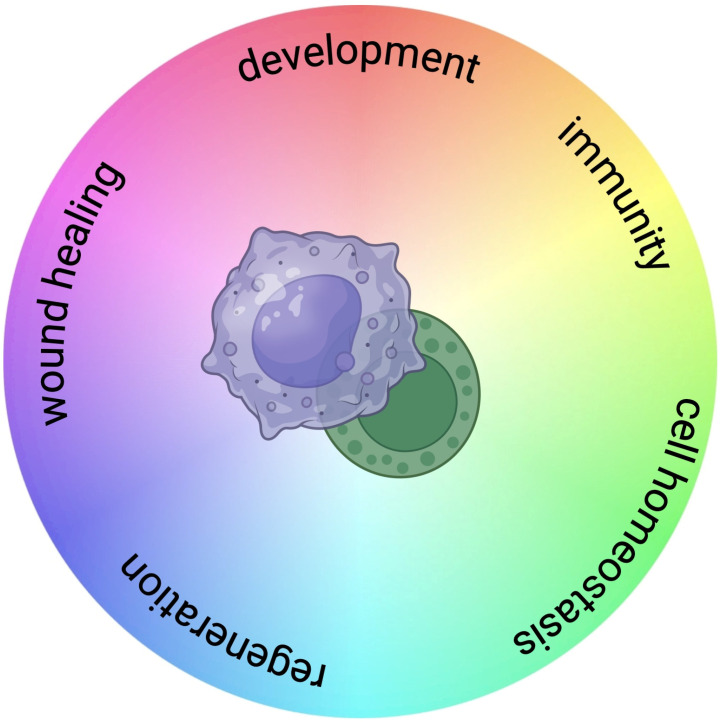
The functional complexity of circulating invertebrate immunocytes. Morphological and molecular evidence has revealed that circulating hemocytes play numerous roles related to immunity and of non-immune-related processes, including development, stress response, wound repair, and regeneration.

More recently, morphological, functional, and molecular evidence has revealed an unexpected complexity of the immune system, and the term immunocyte has been widely used [7,10,11,12,13,14], to refer to circulating and immune-related hemocytes, to emphasize the awareness that circulating hemocytes play numerous roles related to immunity and development, and are not limited to the phagocytic response against microbial pathogens (Figure 1). Immunocyte complexity encompasses the diverse array of immune-associated molecular mediators and receptors produced by immunocytes, as well as the involvement of immunocytes in numerous biological processes, such as development, regeneration, and environmental stress response, that are not related to pathogen-mediated challenges.

## 3. The Functional Diversity of Immunocytes 

An important aspect that has emerged in the last decade is the limitation of morphological classification of immunocytes. Indeed, cells with similar morphology may express specific subsets of mediators, suggesting that immunocyte specialization is more refined than initially thought. As mentioned above, the morphological characterization of immunocytes is the subject of extensive literature [3,15] and will not be repeated here. However, it is important to note that morphological classification usually refers to size, shape, and cytoplasmic granularity. In some cases, the natural color of the immunocyte may also be taken into account.

In the model insect *D. melanogaster*, a highly migratory population of immunocytes was identified in the metamorphosing pupa by combining single-cell transcriptomics and high-resolution microscopy. This population of immunocytes is restricted to the abdominal segments of the pupa and shows distinct morphological features with respect to typical phagocytic immunocytes (i.e., plasmatocytes) of the fruit fly [16]. Other undifferentiated pupal immunocytes were also observed, but these expressed a number of mediators involved in the response to various pathogens, such as bacteria and fungi [16]. In adult flies, plasmatocyte subpopulations, identified by an unsupervised algorithm, drive the systemic response to oxidative stress by activating the Jak/STAT pathway and inducing the cytokine Upd-3 [17]. scRNA-sequencing has also been successfully used to reveal distinct clusters of hemocytes (subpopulations) in several crustacean models. In the shrimps *Litopenaeus vannamei* [18] and *Marsupenaeus japonicus* [19] and in the freshwater crabs *Procambarus clarkii* [20], *Cherax quadricarinatus* [21], and *Pacifastacus leniusculus* [22], this method, which allows the expression analysis of thousands of transcripts, has led to the identification of several marker genes that are expressed specifically in single hemocyte or individual hematopoietic cell types. In this context, in the freshwater crayfish *P. leniusculus*, two different transglutaminases (TGase 1 and 2) are expressed in different hemocyte types, namely TGase 1 in semigranular immunocytes and TGase 2 in granular immunocytes. Notably, only a subset of each immunocyte type expressed the respective TGase [23], suggesting the possibility that the same morphology may mask the existence of cells with different functions and roles [24,25]. Recent studies in *M. japonicus* and *P. clarkii* have shown that in response to viral or bacterial infections, different types of immune-active hemocytes could be observed in relation to specific immune functions. Importantly, only a subpopulation of cells within a group presenting comparable morphology, e.g., macrophage-like hemocytes, could be associated with the expression of specific cell markers and a specific activity (e.g., encapsulation), suggesting that morphological classification alone may be reductive in representing the functional diversity of hemocytes [20].

Further studies in mollusks have confirmed that the complexity observed in Pancrustaceans is not exceptional and should be considered a basic feature of invertebrate immunocytes. Recent experiments using advanced image-based classification have shown that, even in the absence of specific markers, circulating immunocytes of the freshwater gastropod *Pomacea canaliculata* can be grouped into seven clusters [26]. Although consistent with a previous histological classification [27] of immunocytes into two major groups [Group I (GI) and Group II (GII) cells], further subdivided into four major microscopically recognizable populations (blast-like GI cells, intermediate GI cells, agranular GII cells, and granular GII cells), this in-depth analysis revealed the limitations of the usual microscopy-based classification, demonstrated the dynamism of immunocytes, associated morphological features with specific functions (e.g., phagocytosis), and suggested potential differences between *P. canaliculata* immunocyte populations of male and female individuals [26]. Repeated hemolymph withdrawals at 24 h intervals did not significantly alter the balance between immunocyte populations [28], suggesting the existence of mechanisms capable of maintaining a balance between different immunocyte populations. This equilibrium may be fundamental in view of the specific functions that immunocytes may perform outside the circulation. *P. canaliculata* GII granular immunocytes were detected in regenerating tentacle blastema using a specific computer-assisted image analysis protocol [29]. The importance of phagocytic immunocytes for tentacle regeneration was highlighted by the use of clodronate liposomes, which target and temporarily eliminate phagocytic cells. Injection of clodronate delayed tentacle regeneration at a time consistent with the depletion of phagocytic immunocytes. This evidence further suggests that specific immunocyte populations may be associated with different functions, including wound repair and regeneration [30].

## 4. The Molecular Diversity of Immunocytes 

As knowledge of the functional complexity of immunocytes has gradually gained acceptance in the scientific community, so too has knowledge of the humoral component of immunity, which has made tremendous progress in recent decades, driven by the increasing accessibility of sequencing methods and the interpretation of sequenced data. Pioneering studies using immunocytochemical and immunohistochemical techniques, which had the great merit of demonstrating the existence of conserved molecules [31], have gradually been replaced by studies using genome and transcriptome sequences, the latter now feasible at the level of single cells. Some of these latter studies have revealed an extraordinary diversity of molecules and mediators, not necessarily conserved during evolution, and have confirmed the existence of an anticipatory immune system also in invertebrates [32]. In bivalves, for example, the diversity of antimicrobial peptides within the same class reaches extremely high levels, raising the hypothesis that these molecules may not only play a role in the aggression of potential pathogens and the control of the microbiota [33] but also may act as cytokine-like mediators [34].

Examples of some of the best-studied immunocyte-related hypervariable molecules include Down syndrome cell adhesion molecules (Dscams) in insects and crustaceans, fibrinogen-related proteins (FREPs) in mollusks, and Transformer (formerly known as 183-555) in echinoderms. Although evolutionarily unrelated, these hypervariable molecules allow us to define the invertebrate immune system as anticipatory, though not adaptive [32].

The high molecular diversity of the invertebrate immune system was first discovered in *Drosophila melanogaster* [35,36], where the hemocyte-specific loss of Dscam reduced the cell’s ability to phagocytose bacteria, suggesting a potential opsonic role for this hypervariable mediator, for which tens of thousands of isoforms have been reported. The observation that mutually exclusive alternative splicing could generate some 18,000 extracellular receptor isoforms in the larval fat body and hemocytes provided further evidence for the potential of this receptor for immune recognition. Nevertheless, in *D. melanogaster*, Dscam was shown to be a fundamental receptor for sensory neuron branching and connectivity, linking its isoform diversity to neural development rather than immune response [36]. Recent observations have suggested that the functions of Dscam1 isoforms in determining the pattern of axonal branches cannot be fully accommodated within the best-known developmental mechanism based on self-recognition and self-avoidance [37]. Dscam molecules have been discovered and implicated in the immune response in other insects [38], in crustaceans [39,40,41,42] and, more generally, in arthropods [43]; the number of isoforms has led comparative immunologists to hypothesize that Dscam may be involved in immune priming and immune memory [44,45], but to date no conclusive evidence has been reported. One intriguing aspect that remains to be elucidated is whether the presence of hemocyte-specific Dscam isoforms may mask a role for immune-related cells in the development of neural components. In gnathostome vertebrates, the key components of the complement cascade, C1q, C3, and C4, known to be mediators of a fundamental innate immune response, have been implicated in brain development and disease through their role in synapse elimination by marking inappropriate synaptic connections for removal by phagocytic microglia [46,47,48,49]. It would be of great interest to determine whether hemocytes can participate in neural branching in *D. melanogaster*, adding their contribution to the already described mechanisms of neural self-recognition and self-avoidance [37].

First discovered in the freshwater snail *Biomphalaria* glabrata [50], FREPs belong to the class of molecules containing fibrinogen-related domains (FReDs) and are highly diverse lectins [51] that are fundamental to the resistance of the snail in its role of intermediate host of the human parasite, *Schistosoma mansoni* [52]. FREPs molecules contain one or two N-terminal immunoglobulin superfamily (IgSF) domains and a C-terminal FBG-like domain. As a consequence of their structure, different FREPs can bind to different pathogen-associated molecular patterns, or PAMPs [50,53], and one of their fundamental features is that FREPs molecules from different hemocytes of the same individual can differ in their sequence as a consequence of gene conversion and point mutation [54]. In combination with other humoral factors [55,56,57], FREPs diversity is associated with the snail susceptibility to *S. mansoni* infection, as specific FREPs are upregulated only in those strains of snails that are resistant to the Schistosoma infection [52]. While the challenge of *Biomphalaria sp.* snails with *S. mansoni* has provided a fundamental system for modeling trematode–snail interactions and for exploring the basis for specific and hemocyte-mediated immune responses [58,59,60,61,62] in invertebrates, FREPs molecules have also been recovered in other classes of mollusks, such as bivalves [63,64,65,66,67]. In the Pacific oyster, *Crassostrea gigas*, FREPs were among the most up-regulated protein families after exposure for 12 h to different PAMPs, namely lipopolysaccharide (LPS), peptidoglycan (PGN), glucan (GLU), and poly I:C (IC), and were involved in the specific response that varied with time and stimulus applied [63]. The recombinant form of *C. gigas* FREP1 (CgFREP1), designed from a sequence expressed in several tissues, was able to enhance the phagocytic activity of *C. gigas* circulating hemocytes towards the Gram-negative bacterium *Vibrio splendidus*, suggesting a role for this specific isoform in mediating phagocytosis and not only agglutination. Similar to the gastropod *B. glabrata*, the existence of an individual-specific set of FREP sequences has also been reported in bivalves [64,68].

While Dscam and FREPs are representative of molecules diffused in vertebrates and invertebrates, the Transformer (Trf) family, formerly known as 185-333, refers to highly diverse and intrinsically disordered molecules, found only in echinoderms [69,70,71,72]. The restricted diffusion of this family of membrane and soluble receptors has been interpreted as a marker of the dynamism of the invertebrate immune system which may rely on group-specific families of mediators [73]. In this respect, the combined availability of a highly diverse set of immune-related receptors with specific metabolic properties, e.g., anti-oxidant capabilities, may represent an important eco-immunological advantage [74] for maintaining adaptation to different environments, including the adaptation to potential commensals and pathogens. Trf is detectable in both larval and adult sea urchins, and in adults is mainly expressed in specific subsets of coelomocytes, namely polygonal cells and small phagocytes. Each individual contains different Trf molecules [70] and similar to Dscam and FREPs, members of the Trf gene family undergo somatic diversification in single coelomocytes, so that single coelomocytes exhibit significant variation in the Trf gene repertoires [75]. Recombinant forms of Trf exhibited specific binding capabilities, leading to the hypothesis that these molecules underpin the capability of specifically recognizing multiple potential pathogens [72,76], once again defining an immune system with anticipatory features.

Hypervariable molecules seem to confer specific identity and recognition capabilities to individual cells in individual organisms, challenging the notion of a priori excluding the existence of adaptive immunity outside of vertebrates [45,77].

## 5. Immunocytes, Immune Priming, and Trained Immunity

The concept of immune memory in invertebrates, long considered implausible due to the lack of an adaptive immune system, has only recently been confirmed scientifically [78]. Early studies, such as those on *D. melanogaster*, suggested a form of enhanced protection upon re-exposure to pathogens, but it was not until the early 2000s that the phenomenon of immune priming was formally recognized. Landmark works in *Tenebrio molitor* and other insects showed that immunocytes could be primed to mount faster and more robust responses to repeated infections. This discovery shifted the paradigm, suggesting that immune priming may represent an ancient evolutionary strategy to counter pathogenic threats [79,80].

In addition to individual immune priming, which confers cell-specific responses based on previous encounters with pathogens, invertebrates also exhibit transgenerational immune priming (TGIP), a process by which maternal immunocytes transfer immune protection to offspring. In shrimp (*P. monodon*), for example, maternal hemocytes deposit immune signals in the eggs, priming the offspring to resist pathogens encountered by the parent and thus more likely to be present in the environment in which the eggs will hatch. This process gives offspring an immediate advantage in pathogen-rich environments, even in the absence of direct exposure [81]. TGIP has also been documented in insects such as *T. molitor*, where maternal immune priming ensures that larval hemocytes have enhanced antimicrobial activity, particularly against pathogens that posed significant challenges to the previous generation [82].

These findings reveal a fascinating evolutionary continuity. In vertebrates, trained immunity, mediated by innate immune cells such as macrophages and monocytes, represents a functional parallel to immune priming in invertebrates. Trained immunity involves epigenetic and metabolic reprogramming of cells after initial exposure to a pathogen, enabling an enhanced response to subsequent infections. This suggests that the ability of immune cells to take advantage of past encounters may have emerged early in evolutionary history, long before the advent of adaptive immunity. While vertebrates eventually evolved a more specialized adaptive immune system based on antibody-mediated immunological memory, the presence of immune priming and trained immunity in both vertebrates and invertebrates underlines its fundamental importance in survival strategies [83].

From an evolutionary perspective, TGIP in invertebrates and trained immunity in vertebrates may represent complementary solutions to the same problem: ensuring rapid and efficient immune responses in unpredictable and pathogen-dense environments. The conservation of immune priming mechanisms across diverse taxa suggests that this strategy is not merely a substitute for adaptive immunity in invertebrates but an essential and ancient feature of immune defense systems. This evolutionary link emphasizes the shared foundations of immunity across the animal kingdom, providing insights into how immune systems and immunocytes have diversified while retaining core functionalities [81]. In this context, the growing recognition of immune priming and TGIP in invertebrates as forms of innate immune memory broadens our understanding of their immunobiology, and suggests that invertebrate models can provide unique insights into evolutionary and functional aspects of immunity, complementing vertebrate-based studies.

## 6. Non-Immune Roles of Immunocytes: Tissue Regeneration, Development, Homeostasis, and Neuron Turnover

**Figure 2:**
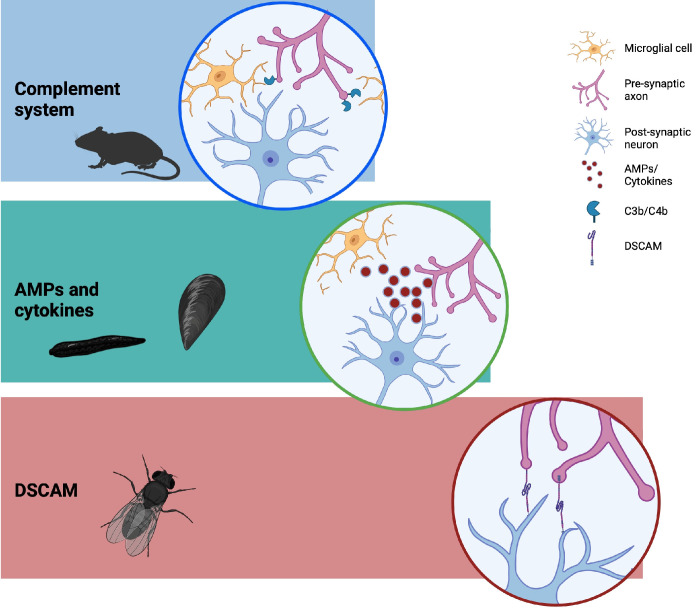
Immune-related molecules involved in homeostasis on neurons and nervous tissue. In phylogenetically distant models, immune-related cells and neurons have been shown to interact via common mediators. Originally discovered for their role in the pathogen-associated immune response, these soluble factors and cell-membrane receptors have subsequently been implicated in neuronal development (e.g., Dscam), synaptic pruning (e.g., complement system components), and immunocyte–neuron interactions (e.g., cytokines).

Studies on the immune functions of immunocytes have progressively unveiled their biological complexity, and parallel research on other biological phenomena has demonstrated their profound involvement beyond the recognition and aggression of potential pathogens. Immunocytes are indeed also pivotal in processes such as regeneration, embryonic development, and neurogenesis (Figure 2).

In *Anemonia viridis*, amoebocytes play a crucial role by migrating to injury sites, releasing antimicrobial compounds, and facilitating the removal of debris, thus ensuring both protection and efficient tissue regeneration [84]. Regarding regeneration, fundamental studies in the leech *Hirudo medicinalis* have shown that the dialogue between the damaged neural component and microglial cells is at the core of the regenerative process [85]. This dialogue is based on evolutionarily conserved neuro-immune molecules, demonstrating how nerve cells can produce mediators normally associated with immune responses to recruit microglial cells, which, in turn, are essential in promoting the regenerative process. The importance of the immune system in the process of neural regeneration in leeches is evidenced by the observations that an experimentally impaired accumulation of microglial cells at the lesion sites resulted in reduced axon sprouting [86]. In addition, microbial challenge can accelerate neural regeneration after axotomy, a process that would involve antimicrobial peptides released by both immune and nervous cells [87].

Data from leeches show that immunocytes do not only play a role in nervous system regeneration. In *Hirudo verbana*, a specific cell type known as telocytes [88] actively contributes to tissue repair by remodeling the extracellular matrix and guiding cell migration through the secretion of HvRNASET2, which also supports fibroblast activation [89]. In addition, the stiffness of the extracellular matrix, which is dynamically modulated during development, has been shown to guide the migration and differentiation of circulating immunocytes, ensuring proper tissue architecture [90]. In the earthworm *Eisenia andrei*, specific immunocyte subsets have been shown to be involved in the regeneration of body segments using specific monoclonal antibodies. Experimental immunocyte depletion in earthworms resulted in a reduced cell proliferation rate in the blastema, confirming the positive role of immunocytes in the regeneration process [91]. As mentioned above, a role for immunocytes in tentacle regeneration has also been hypothesized in the mollusk *P. canaliculata* [29] and has also been proposed in other snails, i.e., *Lymnaea stagnalis* [92] and *Aplysia californica* [93], confirming that the link between immune functions and regenerative processes, known in vertebrates [94,95,96], is widespread in the animal kingdom. In the crayfish *P. clarkii*, the regeneration of the amputated antenna is supported by granular and semigranular immunocytes, whose granules are the source of new cellular organelles (e.g., mitochondria) in the regenerating antenna [97].

Immunocytes are also involved in development and in normal tissue homeostasis and remodeling. In *D. melanogaster*, hemocyte ablation experiments have shown that phagocytic cells are required for the morphogenesis of the central nervous system in embryos [98]. The third hematopoietic wave, which occurs in the larval lymph gland, produces cells that can be involved in immune defense if necessary, but even in the absence of aggression from pathogens or parasitoid wasps, these larval immunocytes may intervene in the removal of obsolete larval components during metamorphosis [99], although the biological relevance of this aspect is debated [24,98,100]. Single-cell RNA sequencing experiments performed on immunocytes from unchallenged *Drosophila* larvae identified cell clusters associated with immune-related processes, such as proliferation, phagocytosis, and humoral response, but also with metabolic homeostasis. Furthermore, the role of immunocytes in tissue development is more relevant in *Drosophila* embryos than in larvae, when the animals are more likely to be exposed to pathogens, confirming the plasticity in the role of these circulating cells [25].

Another example of the great plasticity of the roles of invertebrate immunocytes is the evidence that, in the crustacean *P. clarkii*, first-generation neurons are unable to self-renew and the population of obsolete olfactory neurons is therefore replaced by differentiated hemocytes, that lose their hemocytic properties to become full-fledged olfactory neurons, providing compelling evidence in support of the definition of invertebrate immunocytes as immuno-neuroendocrine cells [101,102]. The production of new neurons is mainly maintained by hyalinocytes, i.e., immunocytes with a hyaline, agranular cytoplasm, and can be influenced by changes in environmental parameters that affect the number of the circulating immunocytes [103,104].

## 7. From the Complexity of the Single Cell to the Development of New Models of Environmental Effects on Immunity

The increased awareness of the complexity of immune cells and their role in immune responses and physiological processes allows the development of invertebrate models for multi-level and transdisciplinary studies, such as the evaluation of the potential impact of the environment on immune functions in animals and humans. Invertebrate models have been proposed for studying the accumulation of micro- and nanomaterials, their cellular-level toxicity, and the interference these xenobiotics can determine with complex processes [105], such as regeneration or wound repair [106,107,108], or gametogenesis [109,110]. This type of study actually offers valuable insights for various research fields including environmental and basic biological research and can find application also to human studies. Recently, the eco-immunological perspective has also been applied for analyzing the possible causes of human illness [111]. The need for an interdisciplinary approach that assesses the impact of pollution on wildlife as well as human health has been identified, but research in this area is still in its infancy [112].

Eco-immunology has been described and summarized [113,114] in different ways because it is a discipline that can be approached from different perspectives [74,83,115,116,117]. On the one hand, the eco-immunological perspective is used to understand how immune functions interact with other bodily functions in order to be balanced in terms of energy efficiency; on the other hand, eco-immunology has also been seen as a translation of laboratory reality into an open field, making variability a point of advantage and study, rather than a parameter to be minimized, as is usually performed in laboratory studies. Eco-immunology has contributed to the understanding of the importance of co-infections in the context of immune response and individual life [118,119], and has gradually made the scientific community more aware of how environmental influences can modify the immune responses of an individual or a population. Many studies in eco-immunology have focused on vertebrate models, but increased understanding of the immune response in invertebrates has led to their adoption for eco-immunological studies. In the bivalve mollusk *Venerupis* (*Ruditapes*) *philippinarum*, it has been observed that immune parameters and biological responses to contaminants are influenced by the sampling site [120]. These observations were made after placing the mollusks in experimental aquaria and controlling the conditions for a period of time, thus demonstrating the importance of environmental imprinting in the context of the immune response. The study of the effects of pollutants on the health status of invertebrate organisms has been applied on many occasions, particularly to cultured mollusks, to monitor the effects of pollution on growth performance and to use the same species as sentinels to assess the pollution status of water. The potential long-term mechanisms of action of high doses of microplastics as carriers of chemical pollutants have been investigated, with microplastic concentrations two orders of magnitude higher than those observed in the Mediterranean Sea and more similar to those of the Californian Current System and the North Pacific Central Gyre [121]. Immunocytes were assessed for immunological changes (lysosomal membrane stability, phagocytosis activity, and granular/agranular cell ratio) and neurotoxic response (enzymatic activity of acetylcholinesterase). The hemocyte immune parameters of lysosomal membrane stability and phagocytosis were both significantly affected by the pollutants, although to different extents depending on the days of exposure. Phagocytosis showed an initial increase as a consequence of exposure to two of the investigated pollutants, whereas it was significantly reduced by long-term exposure (i.e., up to 28 days) to all pollutants investigated. The granular/agranular immunocyte ratio was modified by short-term exposure (7–14 days) to some of the pollutants, whereas no effects were observed at long-term exposure, regardless of the pollutant considered. The multi-variate PCA analysis of the data made it possible to distinguish between physical and chemical effects of the treatments, and although the effects of the treatments were not considered to be pronounced, the sensitivity of the immune system allowed to conclude that they were not negligible. The effects of polystyrene nanoplastics on the lipidomics of mussel immunocytes were also investigated [122]. FIA — (+/− H-ESI) Orbitrap —Exactive analysis of lipid extracts from cultured immunocytes showed that the original lipid composition of the cells was significantly affected by polystyrene nanoparticles, especially those of lower dimensions (50 nm). The changes in the lipid profile indicate a rearrangement of the cell membrane and the oxidation of lipid molecules with a high number of double bonds, which would possibly lead to a reduction in the fluidity of the cell membrane. Although the relationship between this observation and the ability of immunocytes to fight pathogens, as well as the specific susceptibility of immunocyte subsets, remains to be determined, these studies once again demonstrate the level of detail that can now be used to investigate the effects of elusive pollutants, such as micro- and nanoplastics, using immunocytes as a cellular model [123].

## 8. Concluding Remarks

The ensemble of evidence here summarized identifies the immune system of invertebrates as a highly complex biological system with diverse functions, pervasive in numerous aspects of non-pathogen-related organismal life, including development, stress response, wound repair and regeneration. This great complexity, associated with the considerable species-specificity observed for certain molecules or biological functions, might raise the question of whether invertebrates can be a valid alternative to vertebrates in basic studies of immune functions, as widely recommended by animal experimentation regulations. These regulations, starting from the principle of the 3Rs (Replacement, Reduction, Refinement) proposed in 1959 [124], ask researchers to identify research methods that are increasingly ethically less impactful while ensuring effectiveness [125,126,127,128]. In addition, criticisms and perplexities have also been raised regarding the actual utility of mammalian models for immune studies, in the sense that the main mammalian models, such as rodents and lagomorphs, are not always completely reliable in reproducing human disease conditions [129]. In the context of therapeutic development, it should not be overlooked how some recent advancements have been made by bypassing animal experimentation, as the development of personalized therapies in the fight against cancer did not allow for an effective approach to the animal model [130].

While it is undeniable that the increased biological fascination exerted by the complexity of invertebrate immune cells and functions is associated with an understandable difficulty for researchers to obtain data easily translatable from the animal model to our species [131], it is worth noting how increased knowledge of invertebrate immunobiology allows them to be used as comprehensive models for highly diversified research today. The increased understanding of the immunobiological complexity of organisms once considered simple actually allows researchers to identify the most suitable model for the biological question posed by the scientist and has over time contributed to an increase in the number of laboratories using lesser-known organisms as models to study specific biological functions such as hematopoiesis or organ regeneration [132]. This is actually a valuable aspect in the context of animal experimentation because it allows for more complex questions to be asked and for the ethically acceptable and economically sustainable model to be identified to obtain the sought-after answers.

## Data Availability

Not applicable.
